# Long‐acting injectable antipsychotics as maintenance therapy for schizophrenia during the COVID‐19 pandemic: A micro‐narrative review

**DOI:** 10.1002/npr2.12413

**Published:** 2024-01-06

**Authors:** Yoshiyo Oguchi, Nobumi Miyake, Kumiko Ando

**Affiliations:** ^1^ Department of Neuropsychiatry St. Marianna University School of Medicine Kawasaki Kanagawa Japan; ^2^ Department of Neuropsychiatry Kawasaki Municipal Tama Hospital Kawasaki Kanagawa Japan

**Keywords:** COVID‐19, long‐acting injectable antipsychotics, oral medication adherence, psychiatric care, schizophrenia

## Abstract

The coronavirus disease pandemic has presented healthcare systems with unprecedented challenges globally and substantially impacted the management of chronic diseases such as schizophrenia. This narrative review highlights the usefulness of long‐acting injectable antipsychotics (LAIs) as maintenance therapy for patients with schizophrenia during the pandemic. The analysis of relevant literature and psychiatric survey data revealed diverse trends in LAIs prescription and patient adherence with oral antipsychotics. Although some studies have reported a decrease in LAIs prescriptions owing to pandemic‐related disruptions, others have suggested stable patient adherence with oral antipsychotics. Approximately 70% of Japanese psychiatrists reported an increase in schizophrenia relapse rates in a survey, underscoring the critical role of LAIs in maintaining therapeutic stability. The potential benefits of LAIs with extended dosing intervals have been highlighted, including improving oral medication adherence and reducing the frequency of hospital visits. In conclusion, this review emphasizes the continued need for uninterrupted LAIs therapy in conjunction with community and home‐based care despite the disruptions caused by the coronavirus disease pandemic. Further development of LAIs maintenance therapy strategies considering the ongoing pandemic and potential future public health emergencies are required.

## Abstract 2

Evidence of trends in LAI in different countries during the COVID‐19 pandemic.

## 1 INTRODUCTION

Schizophrenia is a chronic illness characterized by frequent relapses and is often exacerbated by non‐adherence to oral medication.^1^ Thus, long‐acting injectable antipsychotics (LAIs) have been considered a promising treatment option. However, empirical evidence supporting their efficacy was limited until recently. A recent meta‐analysis of randomized controlled trials, cohort studies, and mirror image studies showed fewer hospitalizations and relapses among patients treated with LAIs than among those treated with oral antipsychotics, with no significant differences in adverse effects.^2^ In Fiscal Year 2020, Japan, a nation with a long‐standing universal health insurance system, revised its reimbursement procedures to cover LAIs fees for hospitalized patients and thereby promote LAIs use nationwide.^3^ Simultaneously, the ongoing coronavirus disease (COVID‐19) pandemic necessitated an examination of LAIs prescribing trends in Japan and beyond to understand the status of schizophrenia maintenance therapy during this global crisis. This narrative review aims to present the findings of various studies on LAIs prescribing trends during the COVID‐19 pandemic. This review also discusses the significance of LAIs for patients with schizophrenia during the pandemic, their usefulness in conjunction with community social resources, and the potential benefits of extended LAIs dosing intervals.

## 2 METHODS

Relevant studies on LAIs prescriptions during the COVID‐19 pandemic were sourced from PubMed on October 7, 2023, using the following search strategy: (“Long‐acting inject*” or “LAI”) AND (“schizo*”) AND (Infectious Disease [Title/Abstract] OR Epidemic [Title/Abstract] OR Communicable Disease [Title/Abstract] OR COVID‐19 [Title/Abstract]).

Eight of the retrieved studies were not relevant to the topic of this study. 18 were related to LAIs prescriptions during the COVID‐19 pandemic; however, three were reviews for which no parameters were presented; thus, and 15 were ultimately included for in‐depth analysis.

## 3 LAIS PRESCRIPTIONS DURING THE COVID‐19 PANDEMIC

Following the implementation of lockdown measures after the declaration of COVID‐19 as a pandemic by the World Health Organization (WHO) in March 2020, access to several medical facilities was temporarily restricted. Psychiatric facilities also implemented various preventive measures in prescribing LAIs.^4^

### 3.1 LAIs prescribing trends before and after the COVID‐19 pandemic: A decrease in LAI prescriptions

Our review identified four retrospective observational studies, including one mirror‐image study (Table 1). A significant decline in LAIs prescriptions in Romania was observed following the COVID‐19 pandemic declaration on March 11, 2020. This is likely due to the considerable impact COVID‐19 has had on the entire healthcare system, including export restrictions on drugs and supply delays in pharmacies.^5^, ^6^ Recent literature has suggested other factors, such as patient reluctance to leave home and a reduction in the number of injections due to shorter working hours of clinics.^7^ Barlati et al.^4^ reported that the decrease in new LAIs prescriptions in Italian civilian hospitals was related to a switch to oral medications aimed to prevent reduced number of visits during the COVID‐19 pandemic and interactions with drugs administered for severe cases.

**TABLE 1:** LAI prescribing trends before and after the COVID‐19 pandemic (LAI decreasing).

Study	Design	Participants or subjects	Comparison of time periods	Parameter	Results
Ifteni et al., 2020	Observational study	Patients with schizophrenia at a 150‐bed acute‐care public hospital in Romania	Before COVID‐19 pandemic declaration (from December 2019 to February 2020) vs. after COVID‐19 pandemic declaration (March 2020)	The proportion of LAI prescriptions	Reduction of LAI prescriptions (81% for paliperidone, 49% for risperidone LAI, 70% for aripiprazole, and 90% for olanzapine LAI)
Miron et al., 2022	Retrospective mirror‐image study	Participants diagnosed with schizophrenia in the Clinical Hospital of Psychiatry and Neurology Brasov, Romania, a public academic facility with 150 acute patient beds, 3 departments, and round‐the‐clock emergency care	Before COVID‐19 pandemic (from March 11, 2019, to March 11, 2020) vs. during COVID‐19 pandemic (from March 11, 2020, to March 12, 2021)	The proportion of new administration of LAI	A 48.3% decrease in new LAI cases, decreased from 31% to 13% for paliperidone and aripiprazole and from 10% to zero for olanzapine A significant increase from 28% to 73% for risperidone
Barlati et al., 2022	Retrospective observational study	Two hundred thirty‐six participants diagnosed with schizophrenia in 2020 at two outpatient facilities of city hospitals in Italy	A pre‐pandemic reference year (2019) vs. the first year of the pandemic (2020)	The total number of patients receiving LAI treatment New administrations of LAI The number of LAI treatment dropouts	No significant differences were observed between the two periods related to the total number of patients receiving LAI treatment (*p* = 0.890) and number of dropouts (*p* = 0.262); however, a significant decline was observed in new LAI administrations (*p* = 0.022) Decreases from 71 to 66 for haloperidol decanoate, 33–30 for zuclopenthixol decanoate, 18–11 for risperidone Increasing from 64 to 73 for paliperidone 1‐month type, 13–16 for paliperidone 3‐month type, and 34–40 for aripiprazole
Haider et al., 2023	Observational study	Two online surveys (initial: October–November, 2020, *N* = 35; follow‐up: July–September, 2021; *N* = 21)	Initial survey (from October 7, 2020 to November 2, 2020) vs. follow‐up survey (from July 28, 2021 to September 9, 2021)	The number of facilities reporting changes in prescribing	Although 20% of locations reported decreased adherence to LAIs, most sites reported no detrimental effects of the pandemic on drug adherence Eleven sites (31%) reported switching from shorter to longer injection intervals for LAIs during the pandemic, twelve sites (34%) reported switching patients with schizophrenia from LAIs to oral antipsychotic medicines

### 3.2 LAIs prescribing trends before and after the COVID‐19 pandemic: LAIs prescriptions remain stable or increase

Four retrospective observational studies, including one mirror‐image study, suggested that LAIs prescriptions remained stable or increased during the pandemic (Table 2). Studies conducted in the United States (US; Pittsburgh)^8^ and Australia^9^ attributed the stable LAIs prescription rates to thorough infection control measures and successful communication with patients and families regarding the importance of continued LAIs treatment. A Canadian study suggested that LAIs prescriptions continued during the pandemic due to persistent needs, despite the potential selection bias owing to its pharmacy database‐based design.^10^ A survey of psychiatric providers in the United States indicated that LAIs prescriptions were expected to remain stable or increase in 2021.^11^

**TABLE 2:** LAI prescribing trends before and after the COVID‐19 pandemic (LAI unchanged or increasing).

Study	Design	Participants or subjects	Comparison of time periods	Parameter	Results
Gannon et al., 2020	Mirror‐image study	Patients with schizophrenia at the University of Pittsburgh Medical Center Western Psychiatric Hospital in the United States	<Pre‐COVID period 6 weeks> February 1–15, 2020 February 16–29, 2020 March 1–16, 2020 vs. <COVID limits 6 weeks> March 17–31, 2020 April 1–15, 2020 April 16–30, 2020	The number of LAI prescriptions	<Pre‐COVID period 6 weeks> February 1–15, 2020 → 145 February 16–29, 2020 → 112 March 1–16, 2020 → 154 vs. <COVID limits 6 weeks> March 17–31, 2020 → 116 April 1–15, 2020 → 132 April 16–30, 2020 → 122 A decrease of only 10% in the total of data for a 6‐week mirror‐image period covering the pre‐COVID‐19 and COVID‐19 limits. Only four patients receiving LAI treatment requested to be shifted to oral antipsychotic medication
McKee et al., 2021	Retrospective observational study	National and provincial patient‐level longitudinal prescribing data from Canadian retail pharmacies	Pre‐COVID‐19 Comparator Period March–May 2019 versus COVID Awareness Period December 2019–February 2020 versus Primary COVID Escalation Period March–May 2020 versus COVID Maintenance Period June–August 2020 versus Second COVID Escalation Period September–November 2020	Prescribing rates for LAIs, including new starts, discontinuations, and product swaps, based on the national and provincial patient‐level longitudinal prescribing data from Canadian retail pharmacies	Prescribing rates for LAIs, including new starts, discontinuations, and product swaps, were remarkably steady for the study (i.e., no statistically significant differences)
Kisely et al., 2022	Large‐scale retrospective cohort	10% of sample in a pharmaceutical claims database (the standard database administered by the Australian Government for study) from the Department of Human Services Pharmaceutical Benefits Scheme Patients treated for schizophrenia with three or more supplies of oral or long‐acting injectable antipsychotics between June 1, 2015, and May 31, 2020	April–May 2019 vs. April–May 2020 The COVID‐19 lockdown the Australian Government implemented	The quantities of antipsychotics prescribed and dispensed (the supply of LAI antipsychotics from new prescriptions) based on the pharmaceutical claims database	The supply of second‐generation LAI antipsychotics for new prescriptions grew by 0.6%, maintaining the upward trend from the prior years For all years considered, the first‐generation LAI antipsychotic supply for new medications was relatively low in April and May
Zhdanava et al., 2022	Anonymous cross‐sectional double‐blinded online survey	Individual‐level data from US‐based healthcare practitioners with a specialty in psychiatry that included regular LAI prescribers (those who treat one patient with LAIs monthly)	COVID‐19 pandemic 2020 vs. 2021	Prescriber's perspective: Projected LAI prescription rates	Most prescribers (59.8%) claimed that LAI prescribing rates remained the same in 2020. However, prescribers expected an increase (42.9%) or no change (44.1%) in LAI prescription rates in 2021

### 3.3 Parameter changes related to LAIs during the COVID‐19 pandemic

We identified several studies using parameter changes related to LAIs during the COVID‐19 pandemic (Table 3). These studies highlighted that LAIs treatment was associated with the risk of infection, limited clinic access, and increased frequency of LAIs non‐adherence during the COVID‐19 pandemic.^12^, ^13^ To counter these issues, strategies such as switching to oral medications^12^, ^14^, ^15^ and less frequent LAIs administration^12^, ^15^, ^16^ were adopted. Other infection control measures included home visits^12^ and LAIs administration at community pharmacies.^17^ Notably, no difference was observed in the readmission rates between patients taking oral antipsychotics and those receiving LAIs during the pandemic.^18^ However, more LAIs prescribers reported worsened adherence with oral antipsychotic prescriptions and symptom control in patients with schizophrenia during the pandemic.^15^

**TABLE 3:** Parameter changes related to LAI during the COVID‐19 pandemic.

Study	Design	Participants or subjects	Time periods	Parameter	Results
MacLaurin et al., 2021	Observational study	Eighty‐one patients with schizophrenia treated with LAI in the Freedom Trail Clinic of North Suffolk Mental Health Association	From January to May 2020 (COVID‐19 pandemic)	Number of LAI prescriptions	Twenty‐three individuals switched to an outside injector between January and May 2020, with visiting nurses providing injections most of the time. Three patients shifted treatment, and six switched to oral drugs. The clinic's LAI program was continued for the remaining 45 patients. 15 percent (7 patients) of 45 patients shifted to lengthier formulations of their respective LAIs and visited less frequently Concerning the prescribed LAIs, 30 patients were taking aripiprazole, 18 were receiving paliperidone, 12 were receiving haloperidol, three were receiving risperidone, and one received fluphenazine For skipping their injection or having their prescription switched, no patients needed to be admitted to a mental health facility
Miron AA et al., 2022	Observational study	Twenty‐seven individuals with schizophrenia who had been treated with OLZ‐LAI for a long time and remained in remission at Brasov, Romania's Clinical Hospital of Psychiatry and Neurology	From April 2020 to March 2021 (COVID‐19 pandemic)	Proportion of switches from olanzapine‐LAI to oral olanzapine Recurrence rate	Of 27 patients, 21 (77.77%) preferred to convert to oral olanzapine. Only 6 patients continued the long‐acting formulation After switching from olanzapine LAI to OA, 15 patients (71.42%) relapsed during the subsequent 12 months, and 12 were hospitalized at the emergency psychiatric ward
Mascari et al., 2022	4‐month prospective convenience sample study	Patient satisfaction and perceptions of a long‐acting injectable antipsychotic medication administration service in a community‐based pharmacy	COVID‐19 pandemic	Patient satisfaction and perceptions of a long‐acting injectable antipsychotic medication administration service in a community‐based pharmacy	Eighty‐two percent of patients strongly agreed that they felt at ease having this service at the neighborhood pharmacy and that the service's privacy was satisfactory Only 18% of patients strongly agreed that the community‐based pharmacy was close to their place of employment or residence 71 percent of patients who received this service elsewhere strongly agreed that the LAI medication administration service was more convenient than a similar service received elsewhere
Zhdanava et al. 2022 Sep.	Anonymous cross‐sectional double‐blinded online survey	Individual‐level data from US‐based healthcare practitioners (LAI prescribers) with a specialty in psychiatry.	COVID‐19 pandemic	Prescriber's perspective: The proportion of switches from LAI to oral tablets medication adherence symptom control/relapse frequency	Of the 401 LAI prescribers who met survey requirements, 64.6% reported that their LAI prescribing has not changed (update: 19.2%; decrease: 14.0%) The majority of prescribers shifted the same proportion of patients from OAPs to LAIs during the pandemic as in previous practice (65.1%). None shifted to oral antipsychotics (OAP; 63.3%) or LAI formulations with lower frequency of administration (68.1%) Antipsychotic adherence was unchanged for most patients according to 50.1% of LAI prescribers, and treatment was effective for 44.6% based on symptom control and relapse frequency
Forster et al., 2022	Observational study	Electronic medical data of 101 patients with severe mental illness receiving LAI‐As in the United States	COVID‐19 pandemic	Pandemic‐related barriers to LAI‐A adherence and/or changes to LAI‐A medications	For 33% of the patients, pandemic‐related obstacles to LAI‐A adherence and adjustments to LAI‐A prescriptions were documented Within‐participant comparison of an adherence metric generated from electronic health record data showed that the incidence of missed or delayed LAI‐A doses was slightly higher during the pandemic than it was before the pandemic Only 2 of the 13 patients who were interviewed expected that worries about the epidemic would affect their ability to take their medications as prescribed
Masterich et al., 2022	Retrospective study	Participants with delusional conditions, schizophrenia, and illnesses like schizophrenia at the Clinic for Psychiatry of CHC Split	COVID‐19 pandemic	Number of rehospitalizations of patients with schizophrenia	Regarding whether they took oral antipsychotics or LAIs, there was no difference in the number of rehospitalizations of patients with schizophrenia, schizophrenia‐like disorders, and delusional states
Barnett et al., 2023	Descriptive, cross‐sectional study	Patients with schizophrenia receiving PP3M at the time of the survey West London NHS trust	From April to June 2020 (COVID‐19 pandemic)	Patient's perspective: subjective satisfaction and safety	Most of the 46 individuals who participated in the trial said they were satisfied (89.2%) and felt safer (93.5%) after switching to the three‐monthly formulation during the COVID‐19 pandemic Participants emphasized several benefits of switching to PP3M; convenience was the most reported (93.5%), followed by more excellent quality of life (58.7%), reduced stigma (39.1%), and better adherence (28.3%). Additionally, 93.5% of respondents reported no drawbacks, while 6.5% spoke about worsening symptoms or side effects Only one patient stopped using PP3M after a year, and the total number of hospitalizations decreased within that time frame relative to the year before switching

## 4 MAINTENANCE THERAPY FOR SCHIZOPHRENIA USING LAIS DURING THE COVID‐19 PANDEMIC

Despite the prescribing trends during the pandemic, LAIs remained in demand. According to Zhdanava et al., approximately half of LAIs prescribers in the United States observed no changes in oral medication adherence during the pandemic.^15^ Nevertheless, oral medication adherence remained a concern, as highlighted in other studies.^15^, ^19^

In a survey of Japanese psychiatrists, approximately 70% reported an increase in relapses during the pandemic.^20^ Schizophrenia necessitates continuous drug treatment,^1^ and medical interruptions owing to the pandemic potentially resulted in decreased oral antipsychotic prescriptions. This, in turn, negatively impacted oral medication adherence in patients with schizophrenia. Zhdanava et al.^15^ demonstrated that switching from oral medication to LAIs was a strategy to improve antipsychotic oral medication adherence during the pandemic.

Online medical care has grown in popularity; however, our previous study showed that psychiatrists rated the usefulness of online care as low owing to difficulties in evaluating psychopathology and accessibility issues in certain areas such as rural and underpopulated regions.^20^ Because a decrease in LAIs prescriptions was observed during the early stages of the COVID‐19 pandemic because of fewer face‐to‐face consultations, satisfactory access to LAIs became crucial.^21^ In 2020, the American Psychiatric Association recommended that physicians avoid interrupting LAIs treatment for high‐risk patients with schizophrenia.^22^

MacLaurin et al.^12^ cited the availability of community resources to support ongoing treatment as a critical factor in switching to LAIs during the COVID‐19 pandemic. Notably, users of community services and their families who maintained optimal therapeutic relationships with experienced nursing teams received person‐centered, ongoing care even during the COVID‐19 pandemic.^23^

One major issue highlighted by Correll et al. is reduced access to psychiatric care for patients with severe mental illnesses owing to COVID‐19 control restrictions.^21^ To this extent, Moreno et al. proposed a home treatment to improve access to care,^24^ whereas Gannon et al. underscored the importance of regular in‐person visits for LAIs and oral medication adherence for patients and their families.^8^

The pandemic has resulted in increased anxiety and depression in patients with schizophrenia, as noted in a Turkish study.^25^ Therefore, considering LAIs as maintenance therapy options for schizophrenia during the COVID‐19 pandemic has been crucial, not only to prevent patients from isolating themselves or interrupting their visits but also for direct patient treatment through visits.

## 5 OPTIMAL USE OF LAIS DURING THE COVID‐19 PANDEMIC

Based on our survey, psychiatrists deemed LAIs as effective during the COVID‐19 pandemic.^20^ Several previous studies have supported this view, suggesting that LAIs with extended dosing intervals are advantageous.^6^, ^8^, ^12^, ^20^, ^26^ Patients have shared similar views.^16^ Given that the COVID‐19 pandemic is ongoing and considering future global health emergencies, considering LAIs with even longer dosing intervals may be beneficial. This may facilitate the recovery of patients with schizophrenia by improving oral medication adherence and reducing the frequency of hospital visits.

## 6 LIMITATIONS AND FUTURE PERSPECTIVES

This is a narrative review, not a systematic review. Therefore, a systematic review should be conducted in the future. It would be clinically appropriate to examine how LAIs use has changed before and after unforeseen events such as recent wars, earthquakes, and other disasters.

## 7 CONCLUSION

LAIs are essential for maintenance therapy for schizophrenia before and during the COVID‐19 pandemic. While a reduction in LAIs prescriptions was reported during the pandemic owing to distribution issues, some studies showed no significant change. The discrepancies in these findings can be attributed to the variations in the COVID‐19 measures implemented in different regions or settings. Despite these challenges, continued uninterrupted LAIs treatment and community and home care through a multidisciplinary approach are vital in preventing patient isolation, which can exacerbate the symptoms of schizophrenia and pose additional barriers to treatment. Looking forward, the development of new LAIs formulations is eagerly anticipated, as is the continued refinement of LAIs maintenance therapy strategies in the face of unprecedented public health crises such as the COVID‐19 pandemic.

## AUTHOR CONTRIBUTIONS

Y.O., N.M., and K.A. conceived and designed the study. Y.O. drafted the manuscript. All authors have read and approved the final manuscript.

## FUNDING INFORMATION

None.

## CONFLICT OF INTEREST STATEMENT

Dr. Oguchi has received speaker's honoraria from Eisai, Janssen, Kyowa, Otsuka, Sumitomo, Takeda, Lundbeck, Meiji, Mochida, Viatris, and Yoshitomi and has received a scholarship endowment from Eisai. Dr. Miyake declares no conflicts of interest. Dr. Ando has received speaker's honoraria from Janssen, Otsuka, and Sumitomo.

## ETHICS STATEMENT

Approval of the Research Protocol by an Institutional Reviewer Board: N/A.

Informed Consent: N/A.

Registry and the Registration No. of the Study/Trial: N/A.

Animal Studies: N/A.

## Data Availability

Data sharing is not applicable to this article, as no new datasets were generated or analyzed during the current study.
